# Changes in the Characteristics and Initial Treatments of Pulmonary Hypertension Between 2008 and 2020 in Japan

**DOI:** 10.1016/j.jacasi.2022.02.011

**Published:** 2022-05-17

**Authors:** Yuichi Tamura, Hiraku Kumamaru, Takumi Inami, Hiromi Matsubara, Ken-ichi Hirata, Ichizo Tsujino, Rika Suda, Hiroaki Miyata, Shiori Nishimura, Byron Sigel, Masashi Takano, Koichiro Tatsumi, Japan Pulmonary Hypertension Registry (JAPHR) Network

**Affiliations:** a Pulmonary Hypertension Center, International University of Health and Welfare Mita Hospital, Tokyo, Japan; b Department of Healthcare Quality Assessment, Graduate School of Medicine, The University of Tokyo, Tokyo, Japan; c Department of Cardiovascular Medicine, Kyorin University Hospital, Tokyo, Japan; d Department of Clinical Science, National Hospital Organization Okayama Medical Center, Okayama, Japan; e Division of Cardiovascular Medicine Department of Internal Medicine, Kobe University Graduate School of Medicine, Kobe, Japan; f First Department of Medicine, Hokkaido University School of Medicine, Sapporo, Japan; g Department of Respirology, Graduate School of Medicine, Chiba University, Chiba, Japan; h Department of Health Policy and Management, Keio University School of Medicine, Tokyo, Japan; i Medical Affairs, Janssen Pharmaceutical K.K., Tokyo, Japan; j Department of Respirology, Graduate School of Medicine, Chiba University, Chiba, Japan

**Keywords:** Japan, pulmonary arterial hypertension, risk criteria, treatment, 6MWD, 6-minute walk distance, AMED, Agency for Medical Research and Development, BNP, brain natriuretic peptide, CHD-PAH, congenital heart disease–related pulmonary arterial hypertension, CTD-PAH, connective tissue disease–related pulmonary arterial hypertension, ERA, endothelin receptor antagonist, HPAH, hereditary pulmonary arterial hypertension, IPAH, idiopathic pulmonary arterial hypertension, JAPHR, Japan Pulmonary Hypertension Registry, mPAP, mean pulmonary arterial pressure, mRAP, mean right arterial pressure, NO, nitric oxide, NYHA, New York Heart Association, PAH, pulmonary arterial hypertension, PGI_2_, prostacyclin, PH, pulmonary hypertension, PoPH, portopulmonary hypertension, PVR, pulmonary vascular resistance

## Abstract

**Background:**

Pulmonary arterial hypertension (PAH) is a rare, progressive disease. The treatment landscape for PAH in Japan has evolved considerably in recent years, but there is limited knowledge of the changes in treatment practices or patient characteristics.

**Objectives:**

The aim of this study was to evaluate the changes in characteristics and initial treatments for PAH in Japan over time.

**Methods:**

This study used data from the Japan Pulmonary Hypertension Registry (JAPHR) to compare patient characteristics and treatment practices between 2008-2015 (n = 316) and 2016-2020 (n = 315).

**Results:**

The mean ± standard deviation age at diagnosis increased from 47.9 ± 16.7 years in 2008-2015 to 52.7 ± 16.9 years in 2016-2020. The mean pulmonary arterial pressure decreased from 45.4 ± 15.0 to 38.6 ± 13.1 mm Hg. Idiopathic/hereditary PAH was the most common etiology in both periods (50.0% and 51.1%, respectively). The proportion of patients prescribed oral/inhaled combination therapies increased from 47.8% to 57.5%. Oral/inhaled combination therapies were frequently prescribed to patients with congenital heart disease-related PAH (81.8%). There was no significant trend in prescribing practices based on French low-risk criteria: among patients with 0, 1, 2, 3, or 4 criteria, 53.8%, 68.8%, 52.8%, 66.7%, and 39.4% were prescribed oral/inhaled combination therapies, and 0%, 16.7%, 27.0%, 17.3%, and 15.2% were prescribed oral/inhaled monotherapies. Macitentan, tadalafil, selexipag, and epoprostenol were the most frequently prescribed drugs.

**Conclusions:**

The severity of PAH decreased over time in Japan. Oral/inhaled combination therapies were generally preferred. Physicians generally prescribed therapies after considering the patients’ hemodynamics and clinical severity. (Japan Pulmonary Hypertension Registry [JAPHR]; UMIN000026680)

Pulmonary arterial hypertension (PAH) is a rare, progressive disease with a poor prognosis^1^^,^^2^ that has devastating effects on patients in terms of excess disability, financial burden, and impaired quality of life.^3^, ^4^, ^5^ PAH is defined as a sustained elevation of mean pulmonary arterial pressure (mPAP) of ≥25 mm Hg at rest, as measured by right heart catheterization.^6^ Group 1 PAH is further defined as pulmonary artery wedge pressure ≤15 mm Hg and pulmonary vascular resistance (PVR) of >3 WUs in patients without other causes of precapillary pulmonary hypertension (PH) or other rare diseases. The Fifth World Symposium on Pulmonary Arterial Hypertension^7^ classified PAH as idiopathic, heritable (*BMPR2* mutation, other), drug- and toxin-induced, or associated with connective tissue disease (CTD), HIV infection, portal hypertension, congenital heart disease, or schistosomiasis. Estimates suggest that the prevalence of PAH ranges from 10 to 52 cases per million people,^8^, ^9^, ^10^ and it is ∼2 to 4 times more common in female subjects than in male subjects.^11^^,^^12^ Based on reported cases and the population of Japan in 2019, we estimate that the prevalence of PAH in Japan is ∼32 cases per 1 million people.^13^, ^14^, ^15^ Among newly diagnosed patients, results of the REVEAL (Registry to Evaluate Early and Long-Term Pulmonary Arterial Hypertension Disease Management) study in the United States showed that the 5-year survival rate varied according to type; ie, 68% in patients with idiopathic pulmonary arterial hypertension (IPAH) and 47.6% in patients with connective tissue disease-related pulmonary arterial hypertension (CTD-PAH).^16^ The long-term survival was also greater in patients with better functional class at baseline.

Over the last 30 years, the introduction of new drugs such as prostacyclin, endothelin receptor antagonists (ERAs), and phosphodiesterase type 5 inhibitors, as well as the accumulation of demographic and hemodynamic data on PAH, have led to marked improvements in its treatment and prognosis. Indeed, the 5-year survival rate has increased from 34% (95% CI: 24%-44%) for patients diagnosed with PAH between 1981 and 1985^17^ to 61.2% for patients diagnosed with PAH between 2006 and 2009.^16^ Combination therapy may also improve outcomes; eg, in the AMBITION (Ambrisentan and Tadalafil in Patients with Pulmonary Arterial Hypertension) study, the risk of clinical failure in patients with CTD-PAH was 57% lower in patients who received combination therapy than in those who received monotherapy.^18^

After the introduction of novel therapies, the European Society of Cardiology and the European Respiratory Society first introduced guidelines for the clinical management of PAH in 2015.^6^ These guidelines encompass a treatment algorithm advocating monotherapy or combination therapy, depending on the patient’s diagnosis and functional class. The guidelines also recommend combination therapy and management of pediatric and adult patients at expert referral centers. Another novel aspect is the incorporation of a risk stratification mechanism, whereby risk is classified as low, intermediate, or high, and the ultimate goal is to keep patients in the low-risk group. However, the applicability of these cut points is still unknown, and further research is needed. Moreover, the introduction of new drugs has potential real-world implications for patients with PAH. For example, the treatment practices in Japan may have changed after the approvals of macitentan, selexipag, and iloprost in 2015, 2016, and 2015 (launched 2016), respectively.

We therefore sought to investigate the changes in the characteristics and treatment practices for PAH in Japan by examining recent trends in the Japan Pulmonary Hypertension Registry (JAPHR), a network of PH hospitals in Japan. The JAPHR initially involved 8 centers in Japan, with 189 consecutive patients with PAH recruited between 2008 and 2013.^19^ In 2019, the Japan Agency for Medical Research and Development (AMED), the International University of Health and Welfare, and Actelion formed a public–private partnership to analyze data from the JAPHR. This allowed the JAPHR network to expand to 49 institutions with 892 cases by March 2020. This increased sample size will not only allow us to provide further insight into the evolving characteristics of and treatments for PAH but also enhance the external validity of this study.

## Methods

### Ethics

The JAPHR is an industrial–academic–government collaborative study supported by AMED, the International University of Health and Welfare, and Actelion (now Janssen), as a Clinical Innovation Network research program entitled “Research Utilizing the Japan PH Registry to Accelerate Industry-University-Government Cooperation.” The expanded registry was approved by a central Institutional Review Board at Kyoto University Graduate School (Approval No. R1919). Where necessary, approval was also obtained from the ethics committees/institutional review boards at the participating institutions. The academic institutions were in charge of drafting the research project, building the data platform, data management, statistical analysis, communication with study registration sites, and monitoring. The sponsor was responsible for discussions regarding the research project, proposals for data analyses, and funding. The study was registered on the University Hospital Medical Information Network clinical trial registry (UMIN000026680). All patients provided written informed consent.

### Patients

Patients who were diagnosed with PAH at 49 participating institutions according to right heart catheterization examination conducted before January 2020 were registered in the JAPHR between April 2008 and March 2020. As previously explained,^19^ the eligibility criteria were age ≥18 years and diagnosis of PAH after right heart catheterization, with an mPAP ≥25 mm Hg, pulmonary artery wedge pressure ≤15 mm Hg, and PVR >3 WUs. Patients who were not prescribed a pulmonary vasodilator within 6 months after the index visit diagnosis were excluded from the current analyses. The hemodynamic criteria and target population were specified in the study protocol approved before the start of patient enrollment.

**Figure 1:**
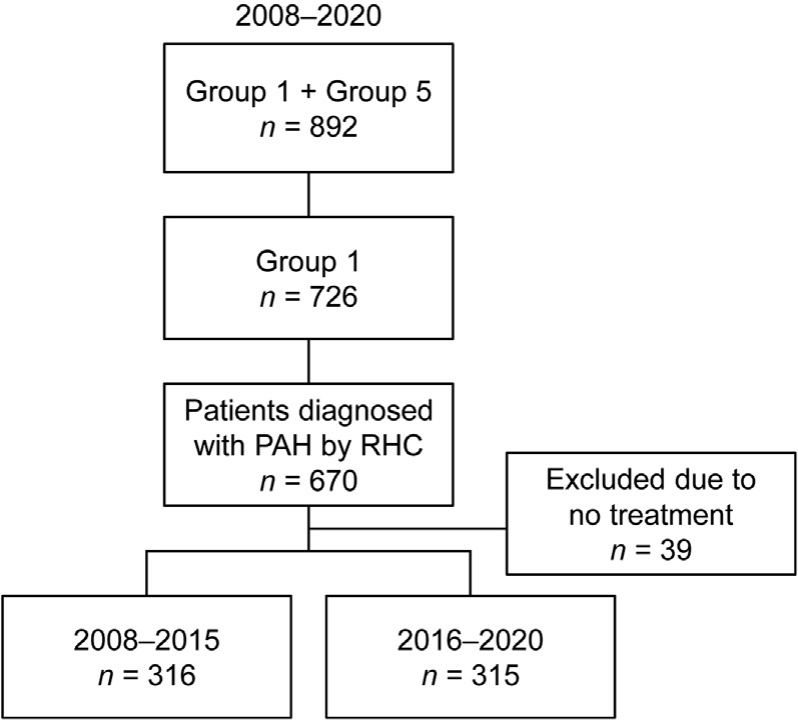
Patient Selection Group 1: pulmonary arterial hypertension (PAH). Group 5: pulmonary hypertension with unclear and/or multifactorial mechanisms.^6^ RHC = right heart catheterization.

Eligible patients were divided into 2 groups depending on when they first visited a participating institution: 2008-2015 or 2016-2020 (Figure 1). These periods were chosen because the treatment landscape in Japan was expected to have changed after the publication of the AMBITION trial in 2015 and the approvals of macitentan, selexipag, and iloprost in 2015, 2016, and 2015, respectively. Laboratory data recorded at the first visit were used as baseline data. The initial treatment was recorded as the treatment used up to 6 months after the initial visit.

### Patient characteristics and treatment patterns

Patient characteristics included New York Heart Association (NYHA) functional class, 6-minute walk distance (6MWD), brain natriuretic peptide (BNP) levels, mPAP, mean right arterial pressure (mRAP), PVR, cardiac index, and mixed venous oxygen saturation. We also assessed the number of low-risk criteria met by each patient based on French criteria definitions 1 and 2.^20^ The etiology was classified as IPAH/hereditary pulmonary arterial hypertension (HPAH), CTD-PAH, congenital heart disease-related pulmonary arterial hypertension (CHD-PAH), portopulmonary hypertension (PoPH), and other. Treatment patterns were classified as oral/inhaled monotherapy, oral/inhaled combination therapy, or parenteral therapy (monotherapy or combined with an oral/inhaled drug), and the following drug types: nitric oxide (NO; sildenafil, tadalafil, and riociguat), ERA (bosentan, ambrisentan, and macitentan), prostacyclin (PGI_2_; oral/inhaled: selexipag, beraprost, and iloprost; intravenous/subcutaneous: treprostinil intravenous, treprostinil subcutaneous, and epoprostenol), and other.

### Statistical analysis

Patient characteristics were summarized descriptively according to study period (2008-2015 and 2016-2020). Characteristics of the 2016-2020 group were also summarized according to treatment patterns. The Cochran-Armitage trend test was used to assess the trends in the proportions of patients in the monotherapy group for each characteristic. Treatment patterns were also evaluated according to time period, with further assessment of the specific drug types used. The assessment of the treatment pattern and specific drug types used was repeated in the 2016-2020 group stratified according to the etiology of PAH. The level of significance was *P <* 0.05 for all tests. SAS version 9.4 (SAS Institute, Inc) was used for data analyses.

## Results

### Subjects

A total of 631 adults with PAH were registered, including 316 in 2008-2015 and 315 in 2016-2020. Most of the patients were women (76.3% in 2008-2015 and 79.4% in 2016-2020). The mean age at diagnosis increased from 47.9 years in 2008-2015 to 52.7 years in 2016-2020.

Although the distribution of IPAH/HPAH and PoPH remained relatively stable, the proportion of patients with CTD-PAH increased slightly from 24.7% to 32.4% between the 2 periods (Table 1). The most common etiology of CTD-PAH was systemic sclerosis in both 2008-2015 (41.0%) and 2016-2020 (53.9%) (Supplemental Table 1).

**Table 1:** Patient Baseline Characteristics According to Study Period (2008-2015 and 2016-2020)

	2008-2015 (n = 316)	2016-2020 (n = 315)
Female	241 (76.3)	250 (79.4)
Age, y	47.9 ± 16.7	52.7 ± 16.9
Etiology		
IPAH/HPAH	158 (50.0)	161 (51.1)
CTD-PAH	78 (24.7)	102 (32.4)
CHD-PAH	41 (13.0)	22 (7.0)
PoPH	29 (9.2)	22 (7.0)
Others	10 (3.2)	8 (2.5)
NYHA functional class		
I	16 (5.1)	27 (8.6)
II	124 (39.2)	151 (47.9)
III	137 (43.4)	119 (37.8)
IV	39 (12.3)	18 (5.7)
6MWD, m	360 ± 123	362 ± 128
BNP, ng/L	213 ± 301	139 ± 230
Hemodynamics		
mPAP, mm Hg	45.4 ± 15.0	38.6 ± 13.1
PAWP, mm Hg	8.5 ± 3.3	8.6 ± 3.5
mRAP, mm Hg	6.2 ± 3.9	6.0 ± 3.9
PVR, dyn·s·cm^−5^	894 ± 602	622 ± 402
Cardiac index, L/min/m^2^	2.56 ± 1.00	2.92 ± 1.02
SvO_2_, %	67.6 ± 9.1	68.1 ± 9.3

Values are n (%) or mean ± SD.

6MWD = 6-minute walk distance; BNP = brain natriuretic peptide; CHD-PAH = congenital heart disease-related pulmonary arterial hypertension; CTD-PAH = connective tissue disease-related pulmonary arterial hypertension; IPAH/HPAH = idiopathic pulmonary arterial hypertension/hereditary pulmonary arterial hypertension; mPAP = mean pulmonary arterial pressure; mRAP = mean right arterial pressure; NYHA = New York Heart Association; PAWP = pulmonary artery wedge pressure; PoPH = portopulmonary hypertension; PVR = pulmonary vascular resistance; SvO_2_ = mixed venous oxygen saturation.

The NYHA functional class and hemodynamic parameters indicate that the severity of PAH decreased between 2008-2015 and 2016-2020, and the proportion of NYHA functional class III to IV patients (high severity) decreased from 55.7% (NYHA functional class III: 43.4%; NYHA functional class IV: 12.3%) to 43.5% (NYHA functional class III: 37.8%; NYHA functional class IV: 5.7%), whereas the proportion of patients with NYHA functional class II increased from 39.2% to 47.9% (Table 1). Baseline laboratory variables and hemodynamics (2008-2015 vs 2016-2020; mean ± SD), including BNP (213 ± 301 ng/L vs 139 ± 230 ng/L), mPAP (45.4 ± 15.0 mm Hg vs 38.6 ± 13.1 mm Hg), and PVR (894 ± 602 dyn·s·cm^−5^ vs 622 ± 402 dyn·s·cm^−5^), were lower in the 2016-2020 group than in the 2008-2015 group.

### Treatment patterns according to study period

Between 2008-2015 and 2016-2020, the proportion of patients prescribed oral/inhaled monotherapy decreased slightly from 23.1% to 20.3% (Central Illustration), whereas oral/inhaled combination therapy increased from 47.8% to 57.5%. Moreover, prescriptions for oral/inhaled and parenteral combination therapies increased from 75.6% (2008-2015) to 79.4% (2016-2020) of patients. For oral/inhaled combination therapy, the most common treatment combination was an ERA plus NO, which accounted for 37.7% of oral/inhaled combination therapies in 2008-2015 and increased to 41.4% in 2016-2020. Furthermore, for oral/inhaled combination therapies, the proportion of patients who received an ERA (regardless of the combination drug) increased from 90.1% to 95.6%, whereas NO remained constant and PGI_2_ decreased from 62.3% to 56.9%. Notably, triple therapy was the most commonly prescribed oral/inhaled combination, increasing from 43.7% in 2008-2015 to 49.2% in 2016-2020. For oral/inhaled monotherapies, the prescription of ERAs increased, NO decreased, and PGI_2_ remained relatively constant. The proportion of patients prescribed parenteral therapies decreased slightly, from 29.1% to 22.2%, between the 2 periods.

**Central Illustration:**
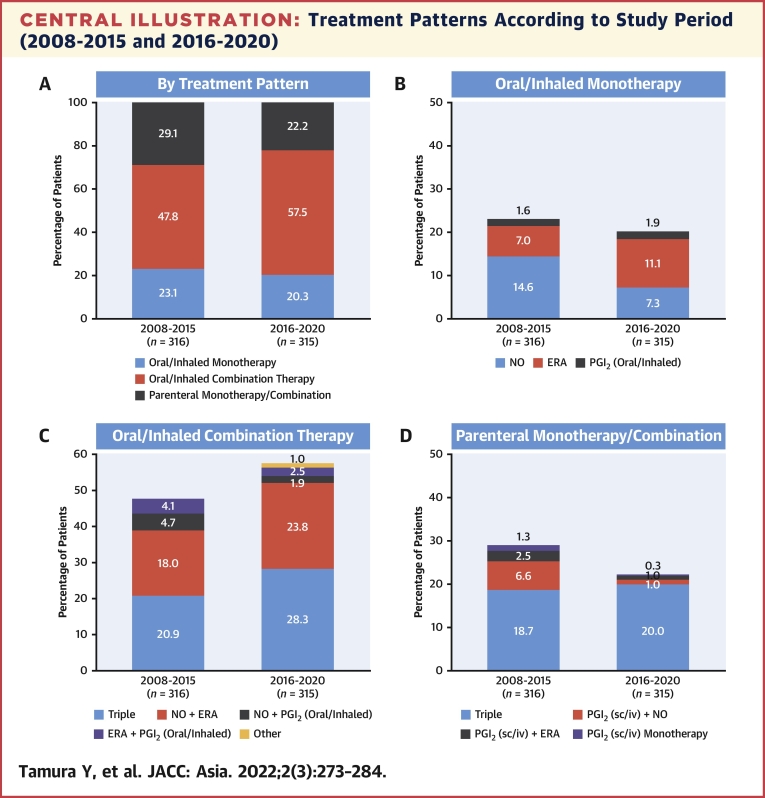
Treatment Patterns According to Study Period (2008-2015 and 2016-2020) Percentages were calculated by using the total number of patients in the time period as the denominator. ERA = endothelin receptor antagonist; iv = intravenous; NO = nitric oxide; PGI_2_ = prostacyclin; sc = subcutaneous.

### Treatment patterns according to etiology in patients diagnosed in 2016-2020

**Figure 2:**
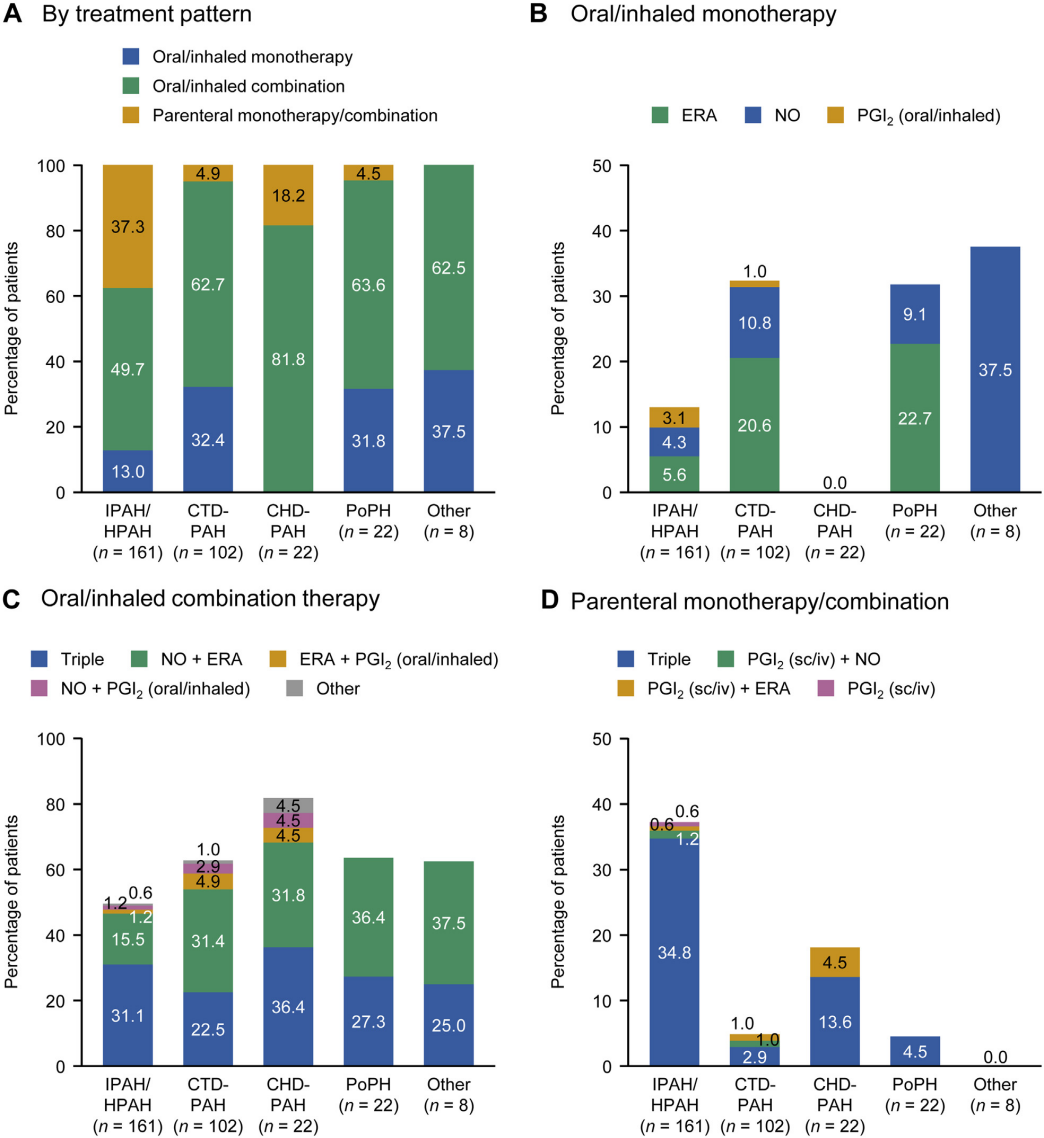
Treatment Patterns According to Etiology for Patients Registered in 2016-2020 Percentages were calculated by using the total number of patients with each diagnosis as the denominator. CHD-PAH = congenital heart disease-related pulmonary arterial hypertension; CTD-PAH = connective tissue disease-related pulmonary arterial hypertension; ERA = endothelin receptor antagonist; IPAH/HPAH = idiopathic pulmonary arterial hypertension/hereditary pulmonary arterial hypertension; iv = intravenous; NO = nitric oxide; PGI_2_ = prostacyclin; PoPH = portopulmonary hypertension; sc = subcutaneous.

The treatment pattern for PAH varied according to etiology. Among patients registered in 2016-2020 (Figure 2), the proportion of patients prescribed oral/inhaled combination therapies was greatest in those with CHD-PAH (81.8%), followed by PoPH (63.6%), CTD-PAH (62.7%), and IPAH/HPAH (49.7%). Less than one-third of patients were prescribed monotherapies. Parenteral therapies (monotherapy or combination) were most frequently prescribed to patients with IPAH/HPAH (37.3%) and CHD-PAH (18.2%).

### Treatment patterns according to clinical assessment, hemodynamics, and risk stratification among patients registered in 2016-2020

Table 2 shows the treatments prescribed in patients stratified into subgroups according to the following baseline risk factors: NYHA functional class, 6MWD, BNP, and hemodynamics (mPAP, mRAP, PVR, cardiac index, and mixed venous oxygen saturation). For NYHA functional class, the proportions of patients prescribed monotherapies, combination therapies, or parenteral therapies were similar between classes I/II and III. None of the patients with NYHA functional class IV were prescribed oral/inhaled monotherapies. Likewise, there were no marked differences in prescribed therapies according to 6MWD or BNP level, except for a high proportion of oral/inhaled combination therapies prescribed to patients with a 6MWD <165 m (70.0%). Regarding hemodynamic factors, there were significant differences in the prescribed therapies among patients divided by mPAP *(P =* 0.003), mRAP *(P =* 0.049), and PVR *(P =* 0.020). For these factors, patients with more severe PAH were more likely to be prescribed oral/inhaled combinations or parenteral therapies. For example, among patients with mPAP *<*30 mm Hg, 31.2% were prescribed oral/inhaled monotherapies and 15.6% were prescribed parenteral therapies; however, among patients with mPAP *>*45 mm Hg, 12.2% were prescribed oral/inhaled monotherapies and 32.9% were prescribed parenteral therapies.

**Table 2:** Characteristics of Patients Registered in 2016-2020 According to Treatment (Excludes Untreated Patients)^a^

	N	Oral/Inhaled Monotherapy	Oral/Inhaled Combination	Parenteral (sc/iv) Monotherapy/Combination	*P* Value
NYHA functional class					
I/II	178	38 (21.3)	100 (56.2)	40 (22.5)	0.44
III	119	26 (21.8)	71 (59.7)	22 (18.5)	
IV	18	0 (0)	10 (55.6)	8 (44.4)	
6MWD					
>440 m	75	10 (13.3)	41 (54.7)	24 (32.0)	0.10
165-440 m	172	39 (22.7)	99 (57.6)	34 (19.8)	
<165 m	20	5 (25.0)	14 (70.0)	1 (5.0)	
0 (missing)	48	10 (20.8)	27 (56.3)	11 (22.9)	
BNP					
<50 ng/L	118	22 (18.6)	62 (52.5)	34 (28.8)	0.88
50-300 ng/L	86	20 (23.3)	47 (54.7)	19 (22.1)	
>300 ng/L	35	6 (17.1)	19 (54.3)	10 (28.6)	
Missing	76	16 (21.1)	53 (69.7)	7 (9.2)	
mPAP					
<30 mm Hg	77	24 (31.2)	41 (53.2)	12 (15.6)	0.003
30-45 mm Hg	155	30 (19.4)	94 (60.6)	31 (20.0)	
>45 mm Hg	82	10 (12.2)	45 (54.9)	27 (32.9)	
Missing	1	0 (0)	1 (100.0)	0 (0)	
mRAP					
<8 mm Hg	215	49 (22.8)	119 (55.3)	47 (21.9)	0.049
8-14 mm Hg	81	11 (13.6)	50 (61.7)	20 (24.7)	
>14 mm Hg	11	1 (9.1)	8 (72.7)	2 (18.2)	
Missing	8	3 (37.5)	4 (50.0)	1 (12.5)	
PVR					
<5 WUs	92	23 (25.0)	51 (55.4)	18 (19.6)	0.020
5-8 WUs	98	24 (24.5)	56 (57.1)	18 (18.4)	
>8 WUs	109	13 (11.9)	65 (59.6)	31 (28.4)	
Missing	16	4 (25.0)	9 (56.3)	3 (18.8)	
Cardiac index					
≥2.5 L/min/m^2^	192	41 (21.4)	109 (56.8)	42 (21.9)	0.24
2.0-2.4 L/min/m^2^	81	17 (21.0)	50 (61.7)	14 (17.3)	
<2.0 L/min/m^2^	36	4 (11.1)	19 (52.8)	13 (36.1)	
Missing	6	2 (33.3)	3 (50.0)	1 (16.7)	
SvO_2_					
>65%	185	33 (17.8)	103 (55.7)	49 (26.5)	0.34
60%-65%	47	11 (23.4)	29 (61.7)	7 (14.9)	
<60%	48	11 (22.9)	27 (56.3)	10 (20.8)	
Missing	35	9 (25.7)	22 (62.9)	4 (11.4)	
No. of low-risk criteria (definition 1)^b^					
4 of 4	33	5 (15.2)	13 (39.4)	15 (45.5)	0.76
3 of 4	75	13 (17.3)	50 (66.7)	12 (16.0)	
2 of 4	89	24 (27.0)	47 (52.8)	18 (20.2)	
1 of 4	48	8 (16.7)	33 (68.8)	7 (14.6)	
0 of 4	13	0 (0)	7 (53.8)	6 (46.2)	
Missing	57	14 (24.6)	31 (54.4)	12 (21.1)	
No. of low-risk criteria (definition 2)^c^					
3 of 3	40	6 (15.0)	17 (42.5)	17 (42.5)	0.40
2 of 3	50	8 (16.0)	30 (60.0)	12 (24.0)	
1 of 3	67	17 (25.4)	38 (56.7)	12 (17.9)	
0 of 3	48	9 (18.8)	25 (52.1)	14 (29.2)	
Missing	110	24 (21.8)	71 (64.5)	15 (13.6)	

iv = intravenous; sc = subcutaneous; other abbreviations as in Table 1.

a Percentages were calculated for each row.

b Definition 1^20^: NYHA functional class I/II, 6MWD >440 m, mRAP *<*8 mm Hg, and cardiac index ≥2.5 L/min/m^2^.

c Definition 2^20^: NYHA functional class I/II, 6MWD >440 m, and BNP *<*50 ng/L.

We further examined if treatment patterns were associated with the ascertained risk derived using definitions 1 and 2 of the French criteria.^20^ When patients were classified according to whether they met any of the 4 low-risk criteria in definition 1 (NYHA functional class I/II, 6MWD >440 m, mRAP *<*8 mm Hg, and cardiac index ≥2.5 L/min/m^2^), oral/inhaled combination therapies were prescribed to 53.8%, 68.8%, 52.8%, 66.7%, and 39.4% of those with 0, 1, 2, 3, or 4 of these criteria. Oral/inhaled monotherapy was prescribed to ≤27.0% of patients in each category. Parenteral therapies (monotherapy or combined with other drugs) were prescribed to 45.5% of patients with 4 low-risk criteria compared with 46.2% of patients with 0 low-risk criteria. Similar findings were observed when we applied definition 2, which comprises 3 criteria (NYHA functional class I/II, 6MWD >440 m, and BNP *<*50 ng/L). The trends for prescription practices were not statistically significant when applying definitions 1 or 2 *(P =* 0.76 and *P =* 0.40, respectively).

### Treatments according to the etiology of pah among patients registered in 2016-2020

**Figure 3:**
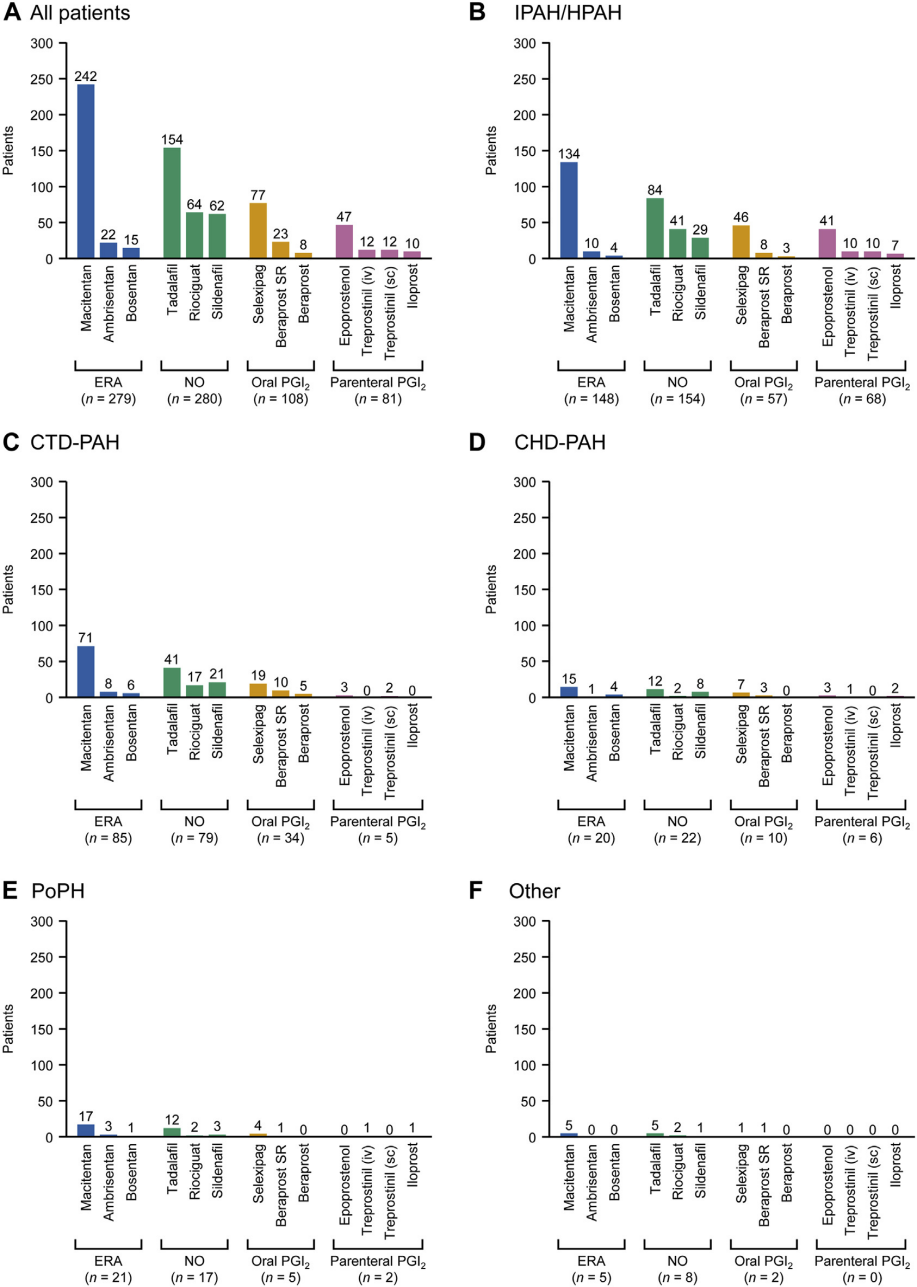
Treatments Prescribed According to Etiology for Patients Registered in 2016-2020 The numbers include all patients who received each type of drug; some patients received multiple therapies and are included in multiple categories. SR = sustained release; other abbreviations as in Figure 2.

Figure 3 shows the number of patients prescribed specific drugs according to the etiology of PAH, regardless of whether they received the drugs as monotherapy or combination therapy. Among patients with IPAH/HPAH, the most common etiology, the most frequently prescribed ERA, NO, oral PGI_2_, and parenteral PGI_2_ were macitentan (90.5% [134 of 148]), tadalafil (54.5% [84 of 154]), selexipag (80.7% [46 of 57]), and epoprostenol (60.3% [41 of 68]), respectively. In patients with CTD-PAH, macitentan (83.5% [71 of 85]), tadalafil (51.9% [41 of 79]), selexipag (55.9% [19 of 34]), and epoprostenol (60.0% [3 of 5]) were the most frequently used drugs in each class, with similar trends in patients with CHD-PAH or PoPH.

## Discussion

This report presents a comprehensive description of the trends in the characteristics and treatment patterns of PAH in Japan. We found that the proportion of patients presenting with severe PAH (NYHA functional class III and IV) decreased between 2008-2015 (55.7%) and 2016-2020 (43.5%). This trend might suggest improvements in medical care, management, and awareness of PAH in Japan. In particular, the 2017 guidelines for PH issued by the Japanese Circulation Society and the Japanese Pulmonary Circulation and Pulmonary Hypertension Society Joint Working Group^21^ probably contributed to the improved treatment of CTD-PAH, particularly in recent years.

We observed an increase in the mean age of patients between the 2 study periods, consistent with prior reports.^22^ This may be caused by later referral to a specialist center and hence older age at registration in this registry, and an increase in the number of IPAH patients with comorbidities, who tend to be elderly.

Our results suggest that Japanese clinicians have a strong preference for prescribing combination therapies (oral or parenteral), because 77.5% of patients in the JAPHR were prescribed combination therapy (oral or parenteral), including 43.9% of patients who were prescribed triple therapy (increasing from 39.6% in 2008-2015 to 48.3% in 2016-2020). These values are relatively high compared with those in other countries. For example, combination therapy was prescribed to 28% of patients in the ASPIRE (Assessing the Spectrum of Pulmonary hypertension Identified at a REferral centre) registry in the United Kingdom^23^ and to 40% of patients in REVEAL,^24^ and dual therapy was prescribed to 29% and triple therapy to 14% in a Swiss registry.^25^ Furthermore, the AMBITION study showed that upfront combination therapy with ambrisentan and tadalafil was superior to monotherapy in treatment-naive patients.^26^ A meta-analysis revealed advantages of combination therapy on clinically relevant outcomes (clinical worsening, 6MWD, mPAP, RAP, and PVR), albeit not mortality.^27^ In light of these results, the 2015 European Society of Cardiology and the European Respiratory Society guidelines recommended combination therapy for adults.^6^ The high rates of combination therapies in Japan could be caused by expertise and experience of the PH centers compared with general practice in Japan because the clinicians at these centers may have greater awareness of the benefits of combination therapy for PAH. We also observed an increase in oral/inhaled combination therapy, from 47.8% in 2008-2015 to 57.5% in 2016-2020. In terms of oral combination therapies, the proportion of patients treated with ERAs has increased. This is at least partly caused by the introduction of macitentan in 2015 in Japan. The OPTIMA (Combination Therapy of Macitentan and Tadalafil in Patients With Newly Diagnosed Pulmonary Arterial Hypertension) multicenter, open-label trial showed that initial combination therapy with macitentan and tadalafil improved hemodynamic function and was well tolerated by adults.^28^ The subsequent launch of selexipag in 2016 and results of GRIPHON (Prostacyclin [PGI_2_] Receptor Agonist in Pulmonary Arterial Hypertension Study)^29^ may explain the greater use of combination therapy and triple therapy in the latter period in our study. Furthermore, we observed a decrease in the proportion of patients prescribed parenteral therapy, possibly caused by the decreased number of severe cases in the latter period.

As might be expected, the treatment patterns for PAH varied according to etiology. In patients with IPAH/HPAH, 49.7% of patients were prescribed oral/inhaled combination therapy. Patients with IPAH/HPAH tend to have severe disease, and many were prescribed parenteral (37.3%) or triple (65.8% as oral or parenteral) therapy in this study. Patients with IPAH/HPAH who were prescribed oral monotherapy could have less severe disease, be elderly, or have comorbidities. As with IPAH, many patients with CHD-PAH present with severe disease, and all patients with CHD-PAH were prescribed combination therapies. Because Eisenmenger disease makes surgery difficult in patients with advanced CHD-PAH,^6^^,^^7^ it is likely that the clinicians at Japanese PH centers were more likely to consider combination therapies for patients with CHD-PAH. By contrast, in other reports of CHD-PAH, ∼50% of patients, or less, were prescribed combination therapies.^30^, ^31^, ^32^, ^33^ For patients with CTD-PAH, the proportion that received combination therapy was slightly lower than that of patients with other etiologies. This scenario is likely caused by the greater proportion of patients with mild symptoms or complications related to systemic sclerosis–associated PAH.^34^ The lower use of combination therapy, therefore, is likely not caused by poor prescription practices but instead may be caused by the limited evidence showing benefits of combination therapy in patients with CTD-PAH. Interestingly, for PoPH, a large proportion of patients were prescribed oral/inhaled combination therapy, which differs from the European and Japanese guidelines.^6^^,^^21^ Most clinical trials to date have excluded patients with PoPH, resulting in limited evidence to support combination therapy, hence the recommendations for monotherapy. However, in this registry, approximately two-thirds of patients with PoPH were prescribed combination therapy (oral or parenteral), suggesting that the clinicians at the participating PH centers recognized that these patients may benefit from combination therapy. Recently, the PORTICO (Portopulmonary Hypertension Treatment With Macitentan—A Randomized Clinical Trial) study^35^ showed that macitentan improved outcomes compared with placebo in a pooled population of patients treated in the context of monotherapy or combination therapy, and there were no differences in adverse events between placebo and macitentan (including combination therapy) for PoPH. Such evidence could have been considered by the clinicians at the Japanese PH centers, especially in the latter period of this study.

In this cohort of Japanese patients, we can see that the prescribing practices varied among patients based on their background characteristics/risk factors. Although there were no significant trends in prescribing practices among patients divided according to NYHA functional class, 6MWD, or BNP level at the index date, we found that oral/inhaled drugs were prescribed as monotherapy to 21.3% of patients with NYHA functional class I/II but not to patients with NYHA functional class IV, and that an oral/inhaled combination was prescribed to the majority of patients with a 6MWD <165 m. We found significant trends for some markers of the severity of PAH, notably mPAP, mRAP, and PVR, because patients with more severe values were more likely to be prescribed an oral/inhaled combination or a parenteral therapy (as monotherapy or in combination with other oral drugs).

We investigated whether prescribing practices at Japanese PH centers were associated with risk stratification criteria using the French definitions. However, we found no significant trends in prescription practices based on definition 1 (ie, NYHA functional class I/II, 6MWD >440 m, mRAP *<*8 mm Hg, and cardiac index ≥2.5 L/min/m^2^) or definition 2 (NYHA functional class I/II, 6MWD >440 m, and BNP *<*50 ng/L).^20^ Oral/inhaled combinations or parenteral therapies were frequently prescribed to patients, regardless of the number of low-risk criteria. The proportion of patients with low-risk criteria increased during the study period. This may be related to improvements in hemodynamic factors, because an earlier analysis of JAPHR showed that improvements in the French risk stratification were associated with improvements in hemodynamic factors.^36^ However, we found no clear relationship between the number of low-risk criteria and prescribing practices. This may imply that Japanese clinicians generally focus on pulmonary hemodynamic values and prefer combination therapies or stronger treatment regimens. In addition, by focusing on the number of low-risk criteria, this scheme takes into account variability in indicators. The reason why nearly one-half of patients with 4 out of 4 or 0 out of 4 low-risk criteria were prescribed a parenteral therapy (45.5% and 46.2%, respectively) is unclear, but it seems likely that the patients with 4 of 4 low-risk criteria were prescribed a parenteral therapy as monotherapy and patients with 0 of 4 criteria were prescribed a parenteral therapy combined with an oral/inhaled drug to manage severe PAH.

Macitentan (86.7%), selexipag (71.3%), epoprostenol (58.0%), and tadalafil (55.0%) were the most frequently prescribed drugs overall and in each of the 4 major classes (ERA, oral PGI_2_, parenteral PGI_2_, and NO, respectively). The similar numbers of patients prescribed an ERA (n = 279) and/or an NO drug (n = 280) indicate that these are the first-choice treatments for PAH in Japan. This pattern was also apparent in patients with IPAH/HPAH and CTD-PAH, the predominant etiologies of PAH. The reasons why macitentan was the most frequently prescribed drug are unclear; however, treatment decisions may take into account its once-daily administration at a fixed dose (no dose adjustment required) and its promising long-term efficacy and safety.^37^ Our data also suggest that it was frequently prescribed in combination with other drugs that can also be administered once daily, particularly tadalafil.^38^

### Study limitations

Because of the study’s retrospective design, the initial follow-up period varied between each center. Nevertheless, the data were monitored to eliminate any inclusion bias. Also, there may be residual confounding between hemodynamic improvements and the treatment choice caused by limitations in adjusting for all conceivable factors when selecting patients for upfront combination therapy. In addition, the hemodynamic function was based on baseline data, and subsequent measures could have been used to determine or modify treatment. Furthermore, because we did not register cases from facilities that only treat a small number of patients, the results may not reflect the practices across Japan. Finally, because the study did not consider the cost of treatment, the data cannot be used to evaluate health economics.

## Conclusions

These findings in a nationwide registry of patients with PAH indicate that its severity has decreased over time in Japan. Oral/inhaled combination therapies were generally preferred for this cohort of patients. The physicians tended to prescribe therapies for PAH taking into account the patients’ hemodynamics and clinical severity.

Perspectives **COMPETENCY IN MEDICAL KNOWLEDGE:** PAH is a rare but serious disease, generally diagnosed based on a sustained elevation of mPAP ≥25 mm Hg at rest, for which there are a variety of treatments. Using data from the JAPHR, we have revealed important trends in the characteristics and initial treatment of PAH in Japan that are relevant to clinical practice.

**COMPETENCY IN PATIENT CARE:** We compared the characteristics of and trends in initial treatment of patients with PAH in Japan between 2 periods (2008-2015 and 2016-2020). The data indicate that physicians generally preferred oral/inhaled combination therapies and that the physicians tended to prescribe therapies for PAH taking into account the patients’ hemodynamics and clinical severity.

**TRANSLATIONAL OUTLOOK:** This study was performed at PH centers treating large numbers of patients. Future studies may provide insight into the clinical practices at smaller or nonspecialist centers.

## Funding Support and Author Disclosures

This study was funded by a Health Labor Sciences Research Grant, Japan and AMED under grant number JP18lk1601003h0001. The funding body contributed to study design and data collection. Editorial support was funded by Janssen Pharmaceutical. Dr Tamura has received remuneration from Janssen and Daiichi Sankyo; research funds from Mochida; and is affiliated with the Pulmonary Hypertension Center, which is supported by an endowment from Nippon Shinyaku. Dr Kumamaru, Dr Miyata, and Ms Nishimura are affiliated with the Department of Health Quality Assessment at the University of Tokyo, a social collaboration department supported by the National Clinical Database, Johnson & Johnson, and Nipro. Dr Miyata is also affiliated with the Department of Health Policy and Management School of Medicine at Keio University that is conducting joint research with the National Clinical Database. Dr Matsubara has received remuneration from Janssen, Bayer, Pfizer Japan, Nippon Shinyaku, Kaneka Medix, GlaxoSmithKline, United Therapeutics, and Mochida; and research funds from Nippon Shinyaku. Dr Hirata has received remuneration from Takeda and Kowa; commissioned, joint, or physician-led research with Daiichi Sankyo, Janssen, Sysmex, and Terumo; scholarship donations from Merck Sharp & Dohme, Janssen, Abbott, Otsuka, Kowa, Sanofi, Takeda, Toa Eiyo, Nippon Shinyaku, Nippon Boehringer Ingelheim, Nihon Medi-Physics, Novartis, Bayer, Biotronik, and Fujifilm Toyama Chemical; and is affiliated with the Division of Cardiovascular Medicine Department of Internal Medicine, which is supported by endowments from Abbott, Medtronic, and Sysmex. Dr Tsujino has received remuneration from Nippon Shinyaku and Janssen; and is affiliated with the First Department of Medicine, which is supported by endowments from Nippon Shinyaku, Nippon Boehringer Ingelheim, and Mochida. Dr Suda is affiliated with the Department of Respirology, which is supported by endowments from Nippon Shinyaku and Janssen. Dr Tatsumi has received remuneration from Janssen. Mr Sigel and Mr Takano are employees of Janssen Pharmaceutical. Dr Inami has reported that he has no relationships relevant to the contents of this paper to disclose.
