# Supplementary material for: Changes in the Characteristics and Initial Treatments of Pulmonary Hypertension Between 2008 and 2020 in Japan
Source: JACC Asia. 2022 May 17;2(3):273–84. doi: 10.1016/j.jacasi.2022.02.011 (PMC9627817; doi:10.1016/j.jacasi.2022.02.011)
Supplement: Supplemental Data [file mmc1.pdf]

Changes in Characteristics and Initial Treatments of Pulmonary Hypertension between 2008 and 2020 in Japan

Appendix: Supplementary Materials

Study investigators

Atsushi Yao (The University of Tokyo Hospital), Kotaro Abe (Kyushu University Hospital), Yoshihiro Fukumoto (Kurume University School of Medicine), Satoshi Ikeda (Nagasaki University Graduate School of Biomedical Sciences), Takahisa Kondo (Nagoya University Hospital), Koichiro Sugimura (Tohoku University Hospital), Yoshihiro Dohi (Kure Kyosai Hospital), Koshin Horimoto (Japanese Red Cross Society Matsuyama Red Cross Hospital), Kazuhiro Kimura (Shinshu University Hospital), Masataka Kuwana (Nippon Medical School Hospital), Masanori Yoshikawa (Nara Medical University), Yoshioki Yamasaki (St. Marianna University School of Medicine), Fusako Sera (Osaka University Graduate School of Medicine), Kaoru Dohi (Mie University Hospital), Masataka Sata (Tokushima University Hospital), Masaru Ishida (Iwate Medical University), Toshiro Shinke (Showa University Hospital), Yasuchika Takeishi (Fukushima Medical University Hospital), Kayoko Kubota (Kagoshima University Graduate School of Medical and Dental Sciences), Naohiko Nakanishi (University Hospital, Kyoto Prefectural University of Medicine), Keiichi Odagiri (Hamamatsu University Hospital), Koichiro Kinugawa (Toyama University Hospital Cardiovascular Center), Naoki Serizawa (Tokyo Women's Medical University), Akiyoshi Hashimoto (Sapporo Medical University), Takeshi Kashimura (Niigata University Graduate School of Medical and Dental Sciences), Koichiro Yoshioka (Tokai University Hospital), Shintaro Nakano (Saitama Medical University International Medical Center), Hakuo Konishi (Juntendo University Hospital), Nobutaka Ikeda (Toho University Ohashi Medical Center), Kazufumi Nakamura (Okayama University Hospital), Masayuki Takamura (Kanazawa University College of Medical, Pharmaceutical and Health Sciences, School of Medicine), and Masaki Ieda (University of Tsukuba Hospital).

Supplementary Table 1. Etiologies of Connective Tissue Disease-Related Pulmonary Arterial Hypertension (2008–2015 and 2016–2020)

**Etiology, n (%)**	**2008–2015 (n = 78)**		**2016–2020 (n = 102)**	
Systemic sclerosis	32	(41.0)	55	(53.9)
MCTD	16	(20.5)	13	(12.7)
SLE	12	(15.4)	9	(8.8)
Overlap syndrome	4	(5.1)	5	(4.9)
Unclassified/other immune disease	14	(17.9)	20	(19.6)

MCTD = mixed connective tissue disease; SLE = systemic lupus erythematosus.
